# Pneumatic Engine

**Published:** 1871-02

**Authors:** 


### PNEUMATIC ENGINE.

This little piece of machinery for Dentist's use has been before the profession for about one year.

We will not attempt to give a description of it here further than to say that it is operated by air put in motion by a foot-bellows. It is so arranged as to give great velocity. It is calculated for various purposes, viz: for running burs for dressing fillings. These are made of various forms and sizes, suitable for all cases to which the dressing bur is applicable. It accomplishes the work with far greater rapidity and much better than can be done by the hand bur, and with far more ease to the operator and the patient. Points can be operated upon, too, with facility that can hardly be reached by the bur in the hand. It will also with equal facility run the polishing cones and wheels of any desirable material. Small burs for opening cavities may be used with great facility. It is arranged so that the bur or drill can be operated at any angle with the shaft of the instrument, thus superseding all the universal drills that have been in use. It is arranged for working files, for dressing fillings, also for carrying slips of wood for polishing.

There is also by the same inventor, Mr. G. F. Green, a pneumatic plugger, in the use of which we have no experience and can not speak as definitely as we would wish. But the bur engine, in its various purposes, we have had in operation for about one year, and have found it so valuable that we could not consent to be without it. T.

---

Order of business for the Twenty seventh Annual Meeting of the Mississippi Valley Dental Society, to be held in Cincinnati, commencing on Wednesday, March 1, 1871:

ORDER OF BUSINESS.

1. Organization, at 10 o'clock, A. M.
2. Calling roll and reading extracts from last annual meeting.
3. Report of officers and paying dues.

Essays and papers relating to the subjects for discussion to be read at the opening of the respective subjects.

DISCUSSIONS.

1. Filling Teeth with Heavy and Light Mallets.
2. Treatment of Soft Tissues of the Mouth.
3. Materials for Filling Teeth.
4. Microscopy of the Dental Tissues.
5. Dental Therapeutics.
6. Anæsthesia.
7. Miscellaneous.

N. W. WILLIAMS,
E. C. SLOAN,
P. G. C. HUNT,
*Committee.*

